# Supplementary material for: Are carbon emissions trading and green financial instruments synergistic? -Comprehensive quantitative research based on content analysis
Source: PLoS One. 2024 Mar 7;19(3):e0298601. doi: 10.1371/journal.pone.0298601 (PMC10919601; doi:10.1371/journal.pone.0298601)
Supplement: S1 Data — (ZIP) [file pone.0298601.s001.zip › Data/地级市Z_F工作报告.zip!/║╙─╧/║╫▒┌╩╨/└┤╫╘╣1⁄2╓┌║┼í╛┬φ┐╦╩2╛▌═°í┐_2014.docx]

各位代表：

　　现在，我代表市人民政府，向大会报告工作，请予审议，并请各位政协委员和其他列席人员提出意见。

　　一、过去五年

　　工作回顾

　　2013年，我市认真贯彻落实党的十八大和十八届二中、三中全会精神及省委、省政府的决策部署，按照市委八届四次全会和市九届人大八次会议的要求，紧抓全省实施粮食生产核心区、中原经济区、郑州航空港经济综合实验区等国家战略规划的机遇，加快构建新型产业基础和新型城市骨架，落实完善一系列政策措施，积极实施10多个专项行动方案，重点打好项目建设、招商引资、企业帮扶三大攻坚战，强化组织领导和要素保障，持续创新，提升发展，经济社会保持良好发展态势。全年地区生产总值同比增长12.5%，规模以上工业增加值增长16%，公共财政预算收入增长21.4%，全社会固定资产投资增长23%，城镇居民人均可支配收入、农民人均纯收入分别增长10.1%和13%，均保持全省先进水平。

　　2013年各项目标的实现，标志着市九届人大确定的任务顺利完成。五年来，面对外部金融危机冲击、自身产业矛盾凸显等错综复杂的经济形势，全市上下团结奋进、开拓创新、务实重干、克难攻坚，各项工作取得明显成效，2013年地区生产总值达到622亿元、公共财政预算收入39.6亿元、城镇居民人均可支配收入21228元、农民人均纯收入10608元，分别是2008年的1.8倍、2.5倍、1.7倍和1.9倍，全社会固定资产投资五年累计完成1870亿元、是上个五年的3.2倍。总体上看，我市经济社会发展站在了新的起点、进入了新的阶段。五年来，我们主要抓了以下工作：

　　（一）稳态势、保增长，经济健康较快发展。坚持把稳态势、保增长放在经济社会发展的突出位置，既坚定信心、统筹协调、保持定力，又主动出击、综合施策、精准发力，及时解决经济运行中的突出矛盾和问题，主要经济指标年度增速保持两位数增长，质量效益得到同步提高。着力抓好经济运行，突出长短结合、标本兼治，完善调节协调机制，优化煤电油气运保障，出台促进工业经济平稳较快增长、工业企业做大做强等措施，实施集中精力战危机、重点打好攻坚战等活动，经济保持平稳健康较快发展。着力激发市场活力，突出市场主体地位，落实税费优惠政策，减轻中小微企业负担，扶持企业做大做强，2013年市场主体户数和注册资本分别是2008年的1.2倍和3.9倍，社会消费品零售总额是2008年的2.1倍。着力深化企业服务，突出健全企业服务长效机制，积极搭建银企对接、用工对接、产销对接等平台，支持企业管理、技术和产品创新，企业发展持续向好，2013年规模以上工业企业增加值、利润分别是2008年的2.1倍和2.9倍。

　　（二）转方式、调结构，两个构建步伐加快。坚持把经济结构战略性调整作为加快转变发展方式的主攻方向，不断优化产业结构和城乡结构。加快构建新型产业基础。突出做强工业，巩固提升煤电化材一体化、汽车及零部件、食品3个特色主导产业，培育壮大金属镁精深加工、电子信息2个新兴先导产业，2013年主导先导产业增加值占规模以上工业增加值的76.2%。继续做优农业，加强农业综合生产能力建设，新建高标准粮田34.4万亩，现代农业产业化集群和新型农业经营主体快速发展，连创25项全国粮食高产纪录，粮食总产实现“十连增”、夏粮实现“十一连增”。努力做大服务业，扎实推进服务业载体和项目建设，文化旅游、商贸物流等提速发展，2013年旅游接待人次、综合收入分别是2008年的2倍和2.2倍，第三产业增加值比2008年增长55.1%。加快构建新型城市骨架。突出“一核双星多支点”单元组合型城市建设，推进城乡一体发展，城镇化率达到53.6%、居全省第3位，城镇绿化覆盖率达到40.2%，被确定为国家智慧城市试点市。130平方公里的城乡一体化示范区获批建设，商务中心区10座标志性建筑基本建成、签约入驻面积超过50%，淇水湾商务休闲区建设全面展开；老城区统筹发展取得重大突破，改造竣工面积350万平方米，解决4.4万户居民住房问题，部分实现集中供暖；县城和重点镇建设有序推进；新型社区建成17个、安置3.7万人。

　　（三）重招商、上项目，发展后劲明显增强。坚持把三位一体推进项目建设、开放招商和产业集聚区建设作为经济社会发展的重点工作，充分发挥拉动支撑作用。突出项目建设主抓手，围绕工业、农业、服务业、社会事业发展和城乡基础设施建设等，共实施重点项目980个，投产379个，完成投资1361亿元，煤化一体化、鹤淇电厂等事关全局的重大项目取得实质性进展。突出开放招商主渠道，围绕主导先导产业，集中资源引龙头、带集群、成产业，共引进超亿元项目643个，实际利用外商直接投资17.3亿美元，引进省外资金653亿元，富士康、航盛、新中源、地恩地、聆海等一批知名企业入驻我市，海关、出入境检验检疫局等口岸平台获批并顺利建设。突出产业集聚区主载体，科学规划建设4个省级产业集聚区和3个市级特色产业园区，目前建成区面积53.2平方公里，累计建设项目830个，一批特色优势产业集群初步形成；鹤壁国家经济技术开发区和以鹤淇产业集聚区为基础的省高新技术产业开发区成功获批，2013年集聚区和园区固定资产投资、规模以上工业企业主营业务收入分别占全市的63.7%、60.1%，已经成为重要的经济增长极。

　　（四）抓改革、促创新，动力活力更加强劲。坚持把改革创新作为应对危机、促进转型、推动科学发展的根本动力，持续积聚发展强大能量。全面促进深化改革，加快转变政府职能、深化机构改革，市级行政审批事项较2008年精简82.5%，精简率居全省前列；扎实推进医药卫生体制改革，市第一、第二人民医院合并重组完成，县级公立医院综合改革顺利实施，在全省率先实现基本药物制度县乡村全覆盖、药品价格下降30%左右；整合成立市文化广电新闻出版局和广播电视台，组建了市豫剧牛派艺术研究院，完成国有文艺院团改革；顺利推进财税、金融、教育、事业单位、户籍制度、城市执法管理体制等改革；完成了煤炭企业兼并重组。全面推动科技创新，累计实施重大科技项目106项，新建省级以上企业研发中心37家、院士工作站2家，获省部级以上科技进步奖11项、其中浚单20项目荣获国家科技进步一等奖，获批创建国家可持续发展实验区、全国质量强市示范市、国家农业科技园区等；2013年科技进步贡献率达到51%、比2008年提高4个百分点，科技综合实力跃居全省第6位。全面破解要素制约，通过对外开拓、对内挖潜，资金、土地、劳动力等要素保障能力明显提升，五年累计融资突破600亿元，落实建设用地7万余亩，完成各类培训超过22万人次，引进了一批急需的高层次高技能人才，其中争取省派科技副职和博士服务团成员数量连续5年居全省第一，较好保障了发展需求。

　　（五）创优势、打品牌，整体形象显著提升。坚持把发挥传统优势、提升比较优势、打造竞争优势作为提升实力和形象的有效举措，努力做精品成特色创品牌。巩固提升现代农业品牌，粮食高产创建、农业标准化和信息化走在全国前列，被确定为全国整建制推进粮食高产创建试点市、全国农业综合标准化示范市、全国整体推进型农业农村信息化示范基地。持续提升循环经济品牌，强化节能降耗，大力推广建筑节能、可再生能源应用，加强污染防治和污染减排，获批创建全国循环经济示范市，被确定为全国建筑节能示范市、国家可再生能源建筑应用示范市。重点提升生态环境品牌，扎实推进以淇河为龙头的生态城市和覆盖全域的林业生态体系建设，淇河保护立法工作取得实质性进展，森林覆盖率提高到31.7%、高出全省8.7个百分点，主要环境指标处于全省先进水平，被确定为全国首批中美低碳生态试点市和省水生态文明建设试点市。强化提升区位交通优势，累计新建改建公路1547公里，公路总里程达到4460公里，公路密度比全省平均水平高55个百分点；石武高铁鹤壁东站、安新高速鹤壁段拓宽工程建成投用，鹤辉高速、晋豫鲁铁路通道顺利推进，形成七纵五横干线公路和十字形高速公路网络，实现从乡到县区、从县区到市半小时交通圈。

　　（六）惠民生、促和谐，人民生活水平不断提高。坚持把保障和改善民生作为出发点和落脚点，力争让人民群众得到更多实惠。五年来累计民生支出262亿元、占财政支出的74.9%，通过市长热线、市长信箱等途径答复解决群众和企业反映诉求超过3.2万件次，一批批重点民生工程如期完成。加强和提升就业和社会保障。新增城镇就业22.4万人，下岗失业人员再就业8.7万人，农村富余劳动力就近就地转移就业率超过40%，被确定为国家创建创业型城市。覆盖城乡的社会保障和救助体系进一步完善，居民健康档案、新农合参合率等工作走在全省、全国前列，企业退休人员养老金、城镇居民医保补助、城乡低保、农村五保等标准不断提高。建设保障性住房11.4万套，竣工或基本建成3.7万套。加强和改善农村生产生活条件。盘石头水库完成移民搬迁1.4万余人、三期移民工作有序推进，完成76个贫困村整村扶贫开发和1.8万户农村危房改造，解决37.6万农村居民及在校师生饮水安全问题，行政村班车通达率100%，2个项目纳入国家或省美丽乡村建设试点。加强和促进社会事业发展。学前教育入学率、义务教育巩固率均居全省前列，普通高招成绩连年提高；职业教育园区建设顺利推进，鹤壁职业技术学院新校区一期投用，新增鹤壁汽车工程职业学院、能源化工职业学院2所专科院校，被评为省职业教育攻坚工作先进市。覆盖城乡的公共文化服务体系初步建立，市艺术中心等一批项目建成投用，成功举办6届民俗文化节；文化产业增加值年均增长15%。城乡医疗和公共卫生服务体系更加完善，爱国卫生、慢性病防治和重大传染病防控等工作扎实开展；低生育水平持续稳定，国家免费孕前优生健康检查实现全覆盖，被列入全国创建幸福家庭活动试点市、全省人口均衡型社会综合配套改革示范市。全民健身活动深入开展，市体育馆建成投用，每年举办沿淇河自行车赛等品牌赛事，竞技体育再创佳绩。老龄、妇女儿童、残疾人和慈善等事业全面发展。加强和创新社会治理。公众安全感、执法满意度居全省前列，被评为全国社会管理综合治理优秀市、法治城市创建工作先进市和全省平安建设先进市、信访工作先进市、安全生产责任目标考核优秀市，社会大局和谐稳定。

　　五年来，我们加强民主法制和精神文明建设，自觉接受人大法律和工作监督、政协民主监督和社会各界监督，办理人大代表建议和政协提案1378件；强化法制宣传和依法治理工作，荣获全国法制宣传教育和依法治理先进市；深入开展文明创建活动，公众道德水平和城乡文明程度进一步提升。加强政府自身建设，行政效能和公务员素质不断提高，依法行政和勤政廉政建设取得新成效。加强国防教育和国防后备力量建设，军民融合式发展取得新进展，军政军民团结不断加强，驻鹤解放军、武警部队和民兵预备役为地方发展作出重要贡献。外事、侨务、对台、民族宗教、统计、史志、档案、气象、地震等工作取得新成绩。南水北调中线工程主体基本完工。对口支援哈密三年任务如期完成。

　　各位代表！过去的五年是我市发展史上很不平凡的五年，经济社会发展取得的成效前所未有，城乡面貌发生的变化前所未有，改革创新推进的力度前所未有，人民群众得到的实惠前所未有，一些工作进入全省全国先进行列、保持较高水平。成绩来之不易，这是省委省政府和市委正确领导的结果，是市人大、市政协大力支持的结果，是各级各部门狠抓落实的结果，是全市人民拼搏奉献的结果。在此，我代表市人民政府，向工作在各条战线上的工人、农民、知识分子、干部、解放军指战员、武警官兵、公安民警致以崇高的敬意，向关心支持我市现代化建设的各界朋友表示诚挚的感谢！

　　在肯定成绩的同时，我们也清醒认识到，当前我市经济社会发展还存在一些突出困难和问题，政府自身建设还存在一些差距和不足：一是经济总量不大，产业结构不优、层次不高、链条不完整的问题没有根本改变，服务业发展相对滞后，支撑带动强的大项目好项目不多，要素约束依然突出，经济转型发展任务艰巨；二是城镇化质量不高，城市骨架已经拉开但尚未形成体系，基础设施、公共服务设施相对薄弱，尤其是老城区和县城功能不完善，城乡一体发展任务艰巨；三是民生“欠账”较多，公共服务体系不够健全，一些方面仍存在薄弱环节，持续提升人民群众幸福指数任务艰巨；四是政府职能转变还不到位，自身建设有待加强，一些工作人员能力素质还不适应新形势新任务要求，还存在作风不实、服务不优、效能不高、为政不廉等问题，提高干部队伍素质和能力水平任务艰巨。对此，我们一定高度重视，认真解决。

　　二、今后五年目标任务

　　党的十八大、十八届三中全会确立了全面建成小康社会和全面深化改革的宏伟目标，省委、省政府勾画了中原崛起河南振兴富民强省的美好蓝图。当前，我市已进入工业化和城镇化加速发展期，正处于克难攻坚、可以大有作为的战略机遇期，既面临十分难得的有利条件，也面临不容乐观的内外环境，我们必须肩负起改革、发展、稳定的责任，进一步坚定信心、抢抓机遇、开拓进取、务实求效，以改革开放的主动赢得发展上的主动，以发展的成果实现富民的目标，奋力夺取全面建成小康社会的新胜利。

　　今后五年政府工作的总体要求是：全面贯彻落实省委、省政府和市委的决策部署，抓住省实施三大国家战略规划机遇，以改革为总统领，以建设生态之城活力之城幸福之城为总方向，以在全省率先全面建成小康社会为总目标，以加快打造两个构建升级版为总任务，着力转方式调结构、稳增长增后劲、创优势强支撑、惠民生促和谐，凝神聚力，实干争先，提升求进，转型发展，不断提高发展质量和效益，提升整体发展水平，促进经济更好较快发展，促进社会大局和谐稳定。

　　到2018年，我们将努力实现以下主要目标任务：

　　综合实力显著增强。力争主要经济指标年均增速保持全省先进水平，地区生产总值年均增长10%以上、总量超过1000亿元，公共财政预算收入超过80亿元、比2013年翻一番，实现经济发展规模、质量和效益同步提高，成为在全省有地位、有实力、有特色、有影响的地区性中心城市。

　　经济结构显著优化。“五带一区”产业格局和“一核双星多支点”城镇化格局更加优化，第三产业增加值占生产总值比重超过25%，高新技术产业增加值占工业增加值比重超过30%，科技进步贡献率达到58%；城镇化率达到60%左右，城镇化质量持续提升，以淇滨区商务中心区、淇水湾商务休闲区为核心的都市功能区综合承载和辐射带动能力明显增强，产兴城兴机制新的活力之城基本建成。

　　生态环境显著改善。努力实现生态改善与产业发展同步，森林覆盖率超过35%；循环经济继续走在全国前列，成为循环经济标准、技术、服务输出市和资源型城市创建循环型城市的典范；圆满完成单位生产总值能耗、主要污染物排放量等约束性目标，天蓝地绿水清的生态之城形象彰显。

　　生活水平显著提高。努力实现居民收入增长和经济发展同步、劳动报酬增长和劳动生产率提高同步，城乡居民人均收入达到全省先进水平；基本公共服务水平和均等化程度全面提高，法治政府、法治社会建设取得积极进展，群众精神文化生活更加丰富，宜居宜业宜游的幸福之城更具魅力。

　　实现上述目标，我们需要把握好以下几项原则：一是坚持一张蓝图绘到底，按照既定的思路目标举措，持之以恒抓落实，一以贯之谋提升。二是坚持改革开放创新，把改革开放创新贯穿于经济社会发展各个领域各个环节，增强改革推动、开放带动、创新驱动能力。三是坚持提高质量效益，把经济增长建立在提质增效可持续发展的基础上，坚持在发展中转型、在转型中发展，注重向质与量同步提升、要素投入与产出更趋合理转变。四是坚持执政为民，把发展经济与改善民生紧密结合起来，让人民群众更多享受改革发展成果。五是坚持务实重干，以实干作风提升各项工作，努力开创改革发展稳定新局面。

　　我们坚信，有省委、省政府和市委的正确领导，有市人大、市政协和社会各界的大力支持，有全市广大干部群众的团结拼搏，在全省率先全面建成小康社会的奋斗目标一定能够如期实现！

　　三、2014年重点工作

　　今年是全面贯彻落实十八届三中全会精神、全面深化改革的开局之年，是实施“十二五”规划的关键一年，是为全面建成小康社会奠定坚实基础的重要一年。做好今年的工作，必须按照市委八届五次全会和经济工作会议的总体部署，坚持“一个主题、两条主线、三个重点、四个着力”的要求，加快转方式调结构，加快打造两个构建升级版，加快提高经济发展质量和效益，加快建设生态之城活力之城幸福之城。

　　今年经济社会发展主要目标是：在提高质量和效益的基础上，生产总值增长10%以上，规模以上工业增加值增长13%以上，公共财政预算收入增长12%以上，全社会固定资产投资增长22%，社会消费品零售总额增长14%左右，城镇居民人均可支配收入、农民人均纯收入均增长10%以上，城镇新增就业3万人以上，人口自然增长率控制在6.5‰以内，居民消费价格涨幅控制在3.5%以内，节能减排完成省下达任务。

　　今年重点做好以下几个方面的工作：

　　（一）抓住关键，持续推进三项重点工作。优化投资结构和方式，提高项目履约开工率、资金到位率、竣工投产率，增强项目拉动、开放带动、载体支撑能力。持续抓好重点项目。集中力量抓好总投资2000多亿元的270个重点项目，力争新开工170个、竣工55个，完成投资430亿元。全力抓好60个重点督导项目，力争煤化一体化项目核准开工，富士康科技园一期建成投产，鹤淇电厂完成投资20亿元。做好项目谋划储备工作，力争新谋划超10亿元项目100个以上。强化扩大开放招商。统筹优化招商力量和资源，注重区域招商和产业招商相结合，大力引进行业龙头、知名品牌、延链补链强链和高科技含量、高成长型项目，严控引进高耗能、高污染、产能过剩的项目，强化落地项目的跟踪服务。打造镁博会、电子信息产业合作洽谈会、食品博览会等特色招商品牌。抓好120个超5亿元的招商项目，引进省外资金、实际利用境外资金、对外贸易出口分别增长15%、10%和10%。积极完善载体平台。加快培育煤化工、食品2个超千亿元和汽车零部件、电子信息、金属镁、纺织服装4个超百亿元特色优势产业集群，促进产业集聚区提质发展，在全省位次明显提升。壮大县域经济，打造发展平台，建成投用海关、出入境检验检疫局，积极申报保税监管中心，做好国家级金属镁出口基地等申报建设工作。完善科技、孵化、检验检测、信息服务等平台建设，提高产业发展支撑能力。

　　（二）突出调整，全力加快产业优化升级。更加注重产业升级和结构调整，在引进龙头、完善配套、扩大规模、提升层次方面下功夫求突破，促进三次产业高水平协调发展。提升做大现代工业。着力抓好总投资1000多亿元的133个工业项目，推动传统主导产业改造升级、连线成面，新兴先导产业做大做强、形成支柱。煤电化材一体化产业，重点发展精细化工，推动产业链向高端延伸，力争开工建设煤制烯烃和石林1号矿等项目，建成聚四氢呋喃、丁二酸酐等项目，努力打造重要的现代煤化工基地和新型陶瓷等特色建材产业集群；食品产业，大力发展高中档面制品和速冻调理、快餐休闲食品，推动向多品种、高品质发展，加快推进50万吨中式营养快餐、6万吨烘焙食品加工等项目，努力打造绿色食品加工基地；汽车及零部件产业，规模化发展改装车、汽车模具、汽车线束，积极推进5万辆专用车、中空成型制造等项目，努力打造龙头支撑有力、产业配套齐全的整体优势，打造改装车和汽模生产基地；电子信息产业，重点发展通讯电子、汽车电子电器，统筹推进光电子、军工电子，加快荣兴科技产业园、合基电讯高新产业园等项目建设，努力打造特色电子信息产业基地；金属镁精深加工产业，重点发展高品质镁合金、镁合金终端产品，加快推进创世电机、明远轻合金等项目，推进国家镁工程中心鹤壁研发中心、镁合金创新工程中心建设，组建河南镁业集团和镁产业技术发展研究院，推动金属镁精深加工与汽车及零部件、电子信息产业融合发展，努力打造“中国镁谷”。同时，扎实推进华塑高分子材料、聆海家居、新亚服装等龙头项目，积极发展高分子材料、现代家居、服饰服装等高成长型制造业。加快发展现代服务业。把服务业作为转方式调结构的战略重点，坚持差异化、特色化发展，扎实做好省服务业综合改革试点工作，依托市商务中心区、特色商业区等载体，加快总投资近600亿元的49个重点服务业项目建设，壮大传统服务业、培育新兴服务业，带动第三产业发展提速、比重提高、水平提升。大力推进休闲旅游业，按照“一区两带四个板块”的总体布局，抓好浚县古城保护与旅游发展、淇河生态区、华漫文化旅游城等一批投资超25亿元项目建设，推动云梦山和古灵山一体创建5A级景区，积极发展文化体验、生态休闲、康体养生等新兴业态，组建旅游发展集团，力求景区建设、服务质量、宣传效果、带动能力有大的提升，努力打造全域式国家级生态文化旅游示范区和全省一流、国内知名的旅游目的地。培育壮大新型物流业，依托宏昌物流、新大陆等重点龙头企业，加快推进各类物流园区和物流网络建设，加快建设豫北煤炭物流储备加工基地和钜桥商贸物流园区，提升煤炭、陶瓷等物流的网络化信息化水平。积极发展电子商务，支持农业硅谷、店连店等搭建电子商务公共服务平台，鼓励大型商贸企业和中小微企业开设网上商城和网店。做精做优现代农业。坚持走在全国前列目标不动摇，深入推进农业结构调整，提升现代农业发展水平。围绕粮食高产创建，实施好国家新增千亿斤粮食生产能力规划工程、中低产田改造、农田水利等基础设施建设，抓好42个小麦、39个玉米高产创建万亩示范片和30万亩高标准粮田示范工程，新建高标准粮田16万亩。壮大现代农业产业化集群，实施好国家农业综合开发现代农业园区等建设，加快发展循环、生态、观光、都市农业，抓好“菜篮子”工程建设；培育龙头企业、专业合作社和家庭农场等，规范有序推进土地流转。提升农业信息和科技水平，抓好农业农村信息化示范基地、农业气象科技专项和国家级农业气象试验站等建设；推进永优浚单玉米种业育繁推一体化发展，打造种业大市。创建全国农业社会化服务综合标准化示范市，提升标准化建设服务水平。加快规模养殖场集约化标准化改造建设，提高畜牧业生态水平和综合效益。

　　（三）注重特色，科学推进新型城镇化。坚持以人为核心，科学规划、产城融合、城乡一体、智能集约、生态宜居，积极稳妥推进新型城镇化，增强与产业发展的相向性、相依性和相容性，全面提升规划建设管理和城乡一体化水平，探索具有鹤壁特色的新型城镇化道路，城镇化率提高2个百分点。加快新型城镇建设。突出中心城区发展，加快都市功能区建设，高水平推进商务中心区、淇水湾商务休闲区、高铁广场片区和开发区电子新城建设，提升辐射带动能力。落实好老城区统筹发展政策措施，以鹤山综合改革实验区等十项建设任务为抓手，提升产业发展、基础设施公共服务设施、生态建设水平，新开工棚户区改造135万平方米，新增集中供热覆盖面积140万平方米。引导县城扩容提质，协调推进旧城改造提升和新城规划发展，推进浚县与中心城区的连接融合和鹤淇一体化，提升集聚产业和人口的能力。稳妥和谐推进美丽乡村试点、重点镇、新型社区和新农村建设。推进城乡一体发展。按照全域鹤壁和城乡统筹的理念，完善城市总体规划、城乡一体化规划和各类专项规划。大胆探索城乡一体化发展体制机制，加快基础设施向农村延伸、公共服务向农村覆盖、现代文明向农村辐射，推进城乡要素平等交换和公共资源均衡配置；落实完善“1+10”政策体系，确保农业转移人口进得来、落得住、转得出。强化综合交通体系建设，抓好晋豫鲁铁路通道、鹤辉高速、鹤浚快速通道、东大线改造等总投资88亿元的12个重点道路交通项目建设，新建农村公路80公里，继续提升公交服务水平；谋划推进与周边城市的城际铁路和交通连接，实现优势互补、融合发展。提高城市功能品位。加快建成区提升，着力解决物业管理、车辆停放、垃圾处理、农贸市场等问题，抓好19个续建和7个新开工城中村的改造工作，提高城市绿化、净化、亮化水平和水电气暖服务保障能力。统筹资源建设智慧鹤壁，同步规划建设城市信息基础设施与市政公用设施、公共服务设施，加快电子政务建设，提升民生和社会领域信息化应用水平，推动“四化”融合发展。注重传承城市历史文脉，融入现代元素，做好城市文化建设，打造精品建筑、精品道路、精品社区。

　　（四）着力提升，积极打造生态环保品牌。始终坚守生态和环保底线，统筹推进生态产业发展、生态环境保护和生态文化建设，加快绿色发展循环发展低碳发展，推动生态文明和美丽鹤壁建设。提升生态建设水平。加快以“三廊三带三区”为骨架、覆盖全域的林业生态体系建设，完成造林绿化20万亩，力争成功创建国家森林城市。积极推进淇河保护立法工作，抓好淇河湿地区和风貌带、淇河流域环境治理及生态保护项目，扎实推进盘石头水库工程验收、移民收尾和生态保护工作，确保南水北调鹤壁段具备通水条件，提升水生态文明试点市水平。启动国家和省级园林城市创建工作。做好矿山地质环境治理。促进循环经济发展。围绕建设国家循环经济示范市，改造提升循环型产业集聚区和园区，力争20个循环经济续建项目11个竣工投产，确保15个循环经济项目开工建设。提升循环经济层次和标准化水平，争取更多标准上升为国家或行业标准，继续走在全国前列。提高节能减排实效。做好国家节能减排财政政策综合示范市创建和中美低碳生态试点市工作，建设一批低碳生态社区和绿色建筑，加快推进13个节能项目建设，强化重点耗能企业节能管理。高标准实施蓝天工程、碧水工程和乡村清洁工程，抓好城市大气污染监测治理和机动车环保管理、河流清洁和饮用水源保护、土壤保护和农村环境综合整治，优化环境容量，提高环境质量，建设清洁家园、清洁水源、清洁田园。做好总量减排、污染防治、环评准入、环境执法等，倡导绿色消费理念和生活方式，引导公众参与生态文明建设，保持主要环境指标在全省的先进水平。

　　（五）深化改革，不断增强发展内生动力。坚持从最有条件、最能见效的领域入手，从群众最期盼的领域、制约经济社会发展最突出的问题改起，看准一件做一件，成熟一项推一项，确保取得实实在在的成效。大力推进改革。以经济体制改革为重点，统筹推进其他领域改革。完成市县政府机构改革任务，稳妥推进事业单位、道路运输管理体制、行政执法体制和工商登记制度改革。扎实推进财税体制改革，优化市县区财政体制；深化政府和部门预决算公开，改进年度预算管理方式，逐步建立跨年度预算平衡机制；清理整合专项转移支付项目，统筹高效使用专项资金；加强政府债务管理，提升投融资平台公司投融资能力、资产管理和风险防范能力；加大向社会购买公共服务力度。深化医药卫生体制改革，抓好公立医院改革发展，健全基层卫生服务体系，大力发展特色民营专科医疗机构，提高公共卫生和基本医疗服务水平。积极推进文化体制机制改革，引导和鼓励社会力量参与公共文化服务和文化产业发展。创新完善产业发展、项目建设、开放招商、行政服务管理等体制机制、方式方法。统筹推进农村、资源性产品价格、土地管理、金融、科技、教育等改革。加强科技创新。扎实推进国家可持续发展实验区、高新技术开发区和农业科技园区建设，深入推进科技创新三年计划和国家粮食丰产科技工程，加强与高等院校、科研院所的合作。依托产业链部署创新链，集中力量支持一批科技创新团队，新建一批产业技术创新战略联盟，实施一批重大科技专项，突破一批关键核心技术和共性技术，新建创新型试点企业和市级以上工程技术研究中心各10家，力争高新技术产业增加值增长15%左右、占工业增加值的28%以上，增强科技创新支撑引领能力。扎实开展全国质量强市示范市创建工作，大力实施商标战略，加强知识产权运用和保护，推进质量提升和品牌建设。深化企业服务。把引进外来企业和壮大现有企业更好地结合起来，建设市企业服务发展中心，设立服务企业的“项目池、技术池、资金池”，健全企业服务长效机制。全面落实鼓励支持引导民营经济发展的政策措施，健全民营企业发展激励机制，积极扶持龙头企业和高成长型企业，大力发展“专精特新”中小企业，促进企业做大做强。做好要素保障。完善金融服务体系，充分利用多层次资本市场、多种金融产品，推进市城投债二期发行和企业上市工作；加快推进农商行挂牌、鹤壁银行改革重组、村镇银行组建和域外股份制银行引进工作；加快发展民生金融，积极开展“助保贷”业务，支持设立创业投资基金和产业投资基金，破解中小微企业、“三农”、小额担保等贷款难题；加强行业监管，有效防范区域性金融风险，维护良好金融生态环境。实施最严格的耕地保护制度和节约集约用地制度，做好低效用地再开发、闲置土地治理等工作，提高建设项目的投资强度、建设进度、综合效益；加快农村集体建设用地和宅基地确权登记颁证工作，推进人地挂钩试点，完善土地供应政策，确保综合可用土地不少于1.7万亩。围绕扩充总量、优化结构，大力实施全民技能振兴工程，重视农民工职业培训和素质提高，推动职业教育特色化、品牌化发展；抓好各类人才队伍建设，推进紧缺人才引进、高层次人才柔性引进、大学生回归等计划，持续提高对人才的吸引力，保障经济社会发展需求。

　　（六）改善民生，切实提高群众生活水平。注重发展经济和改善民生良性循环，加快民生领域基础设施、综合保障体系建设，实施好省市重点民生工程，倾情尽力为人民群众谋利益。增加城乡居民收入。深化收入分配制度改革，推动企业工资集体协商，适时调整最低工资标准，提高农民经营性收入和在土地增值收益中的分配比例，增加城乡居民财产性收入，提高消费能力。完成8个贫困村整村扶贫开发。推动更高质量就业。完善多渠道增加就业政策措施和就业创业公共服务体系，扩大农村劳动力就近就地就业，统筹做好高校毕业生、农村转移劳动力、下岗失业人员就业工作，加大困难就业群体就业帮扶力度，完成各类职业技能培训3万人次以上，农村劳动力转移就业2万人，动态消除零就业家庭；维护劳动者合法权益，构建和谐劳动关系。办好人民满意的教育。完善基本公共教育基础设施和服务体系，优化调整中小学校布局，扩容整合优质教育资源，推进教育资源向农村倾斜，提升城乡教育教学质量。开工建设示范性综合实践基地和两所中小学校，实施8所市区中小学和8所农村义务教育薄弱学校改扩建，加快农村中小学校标准化建设，推进义务教育均衡公平发展。继续实施职业教育攻坚，做好鹤壁能源化工职业学院和汽车工程职业学院招生办学工作，基本建成鹤壁职业技术学院新校区二期，推进特色院校和品牌示范院校建设。启动二期学前教育三年行动计划。抓好民办和特殊教育发展。做好困难家庭学生资助工作。促进文化发展繁荣。大力培育社会主义核心价值体系，强化公民道德教育，深化文明创建活动和诚信建设，培育形成良好社会风尚。加强公共文化设施建设，完善公共文化服务体系，办好民俗文化节等特色文化活动和文化惠民工程，做好文化遗产保护，提升文化服务质量效果，丰富群众文化生活，发扬光大牛派艺术等文化品牌。加快文化改革发展试验区和产业园区建设，举办常态化的文博会展、文化体育等活动，推动文化产业规模化、专业化、特色化发展。加强网络文化建设管理，持续“扫黄打非”，抵制低俗现象。提高健康服务水平。整合优化资源配置，提升公共医疗机构服务水平，建成市人民医院主病房楼、市中医院病房楼，推进“三甲”医院创建工作，支持市中医院、京立医院等与市外医疗机构合作，持续提高办医水平；加快城市社区卫生服务中心建设；积极开展国家卫生城市创建工作。认真落实计划生育政策，提高优生优育水平，促进人口长期均衡发展，深入推进建设全国幸福家庭活动试点市等工作。大力开展全民健身活动，加快市全民健身活动中心等城乡体育设施建设。解决6.3万农村居民和在校师生安全饮水问题。拓宽住房保障渠道。抓好保障性安居工程建设，新开工15542套、基本建成16010套，积极推进廉租房、公租房并轨运行，健全保障房建设、分配、管理、运营机制，逐步解决城镇住房困难家庭、新就业无房职工的住房困难，多渠道解决城镇外来务工人员住房困难；促进房地产业持续健康发展，满足居民多层次住房需求；完成农村危房改造3000户。增强社会保障能力。继续扩大社会保障覆盖范围，企业退休人员基本养老金提高10%，研究城镇职工基本养老保险与城乡居民养老保险衔接办法，探索城镇职工医保与居民医保、新农合的衔接，人均基本公共卫生服务经费标准提高到35元，城镇居民医保和新农合人均财政补助标准提高到320元。健全社会救助制度，落实好社会救助和保障标准与物价上涨联动挂钩机制，保障困难弱势特殊群体基本生活。完善社会养老服务体系，大力发展养老服务业，推进乡镇敬老院改造、老年公寓等建设。积极发展社会福利和慈善事业。做好优抚安置工作。大力发展残疾人、妇女儿童、红十字等事业，开工建设市综合活动中心。关爱留守儿童、留守妇女和留守老人。做好应急避难避险场所、烈士陵园、殡仪馆等建设。加强和创新社会治理。扎实推进平安鹤壁、法治鹤壁建设，重视各类社会组织和基层基础建设，加强和改进信访工作，有效排查化解社会矛盾，强化应急管理体系建设。抓好重点行业重点领域安全隐患排查治理，高度重视安全生产、食品药品安全，坚决遏制重特大事故发生。强化社会管理综合治理，依法打击违法犯罪活动，让社会充满活力、和谐有序。

　　加强国防教育和后备力量建设，支持驻鹤解放军、武警部队、民兵预备役和人民防空建设，深入开展双拥共建，大力发展军民结合产业，促进军民融合深度发展。支持工会、共青团、妇联、工商联等人民团体发挥桥梁纽带作用。做好外事、侨务、对台、民族宗教、统计、史志、档案、地震等工作。做好第三次经济普查、第八届村委会换届选举工作。

　　四、加强政府自身建设

　　认真开展党的群众路线教育实践活动，深化“改作风转职能优环境”活动，以群众和企业满意为标准提升服务质量，以群众和社会需求为导向配置服务产品，持续提高工作水平和服务能力，加快建设为民务实清廉政府。

　　加快转变政府职能。大力简政放权，加强行政审批事中事后监管，切实做到“放”“管”结合，对保留的行政审批事项优化规范流程、全面提高效率；整合社会投诉资源，统筹发挥市行政服务中心、12345行政服务热线等作用，提高行政服务效率和水平。切实转变职能，强化政府公共服务、市场监管、社会管理、环境保护等职责，加快向创造良好发展环境、提供优质公共服务、维护社会公平正义转变。提升能力素质，加强学习型机关建设，提高政府系统工作人员的战略思维、创新思维、底线思维能力。严控机构编制，确保财政供养人员只减不增，推动机构编制资源向重点领域、部门和岗位倾斜，建设服务型政府。

　　深入推进依法行政。坚持用法治思维和方式调节经济运行、管理社会事务，严格依照法定权限和程序行使权力、履行职责，深入推行服务型执法，做到规范公正文明执法。坚决执行人大及其常委会的决议决定，支持政协参政议政，自觉接受人大法律和工作监督、政协民主监督，认真办理人大代表建议和政协提案，广泛听取民主党派、工商联、无党派人士和人民团体的意见。提高决策水平，完善重大决策合法性审查和风险评估机制，确保决策民主、程序正当、结果公开。深化政务公开，对涉及群众利益的事做到公开透明、公正公平，保障群众知情权、参与权、监督权。加强行政监察和审计监督，接受社会和舆论监督，让权力在阳光下运行，建设法治型政府。

　　切实改进工作作风。健全改进工作作风、密切联系群众的长效机制，持续解决“四风”问题，使优良作风成为政府工作人员的自觉行动。坚持人民至上，从让人民群众满意的事情做起，从人民群众不满意的问题改起，做到对群众深恶痛绝的事零容忍、对群众急需急盼的事零懈怠。坚持务实重干，始终保持良好精神状态，增强自信心、进取心、责任心，做到讲实话、办实事、出实绩，提升政府执行力和公信力；持续抓好工作落实、改进文风会风、完善服务方式，进一步提高工作效能，尤其是对争取到的国家、省试点示范工作和市里制定的规划计划方案等，自觉围绕目标要求、务求实际效果、注重创新提升、助推全市发展。坚持廉洁从政，按照教育从实、制度从细、监督从紧、惩处从严的要求，落实完善厉行节约反对浪费各项规定，严控一般性支出，确保“三公”经费只减不增，在政府过紧日子中让人民过上好日子；强化纠风和专项治理，坚决查处损害群众利益行为，坚决查处腐败案件和腐败分子，以反腐倡廉实效保障发展、取信于民，建设廉洁型政府。

　　各位代表！新起点开创了新征程，新阶段肩负着新使命。让我们继续解放思想、改革创新，提升求进、奋发有为，努力迈出建设生态之城活力之城幸福之城新步伐，奋力夺取在全省率先全面建成小康社会的新胜利，为中原崛起河南振兴富民强省作出新的更大贡献！
